# Humanized Mice Engrafted With Human HSC Only or HSC and Thymus Support Comparable HIV-1 Replication, Immunopathology, and Responses to ART and Immune Therapy

**DOI:** 10.3389/fimmu.2018.00817

**Published:** 2018-04-19

**Authors:** Liang Cheng, Jianping Ma, Guangming Li, Lishan Su

**Affiliations:** ^1^Lineberger Comprehensive Cancer Center, University of North Carolina at Chapel Hill, Chapel Hill, NC, United States; ^2^Department of Microbiology and Immunology, University of North Carolina at Chapel Hill, Chapel Hill, NC, United States

**Keywords:** humanized mice, NRG-hu HSC, NRG-hu Thy/HSC, HIV-1 replication, HIV-1 immunopathology, combined antiretroviral therapy, HIV-1 immune therapy

## Abstract

Immunodeficient mice reconstituted with human immune tissues and cells (humanized mice) are relevant and robust models for the study of HIV-1 infection, immunopathogenesis, and therapy. In this study, we performed a comprehensive comparison of human immune reconstitution and HIV-1 infection, immunopathogenesis and therapy between immunodeficient NOD/Rag2^−/−^/γ_c_^−/−^ (NRG) mice transplanted with human HSCs (NRG-hu HSC) and mice transplanted with HSCs and thymus fragments (NRG-hu Thy/HSC) from the same donors. We found that similar human lymphoid and myeloid lineages were reconstituted in NRG-hu HSC and NRG-hu Thy/HSC mice, with human T cells more predominantly reconstituted in NRG-hu Thy/HSC mice, while NRG-hu HSC mice supported more human B cells and myeloid cells reconstitution. HIV-1 replicated similarly and induced similar T cell depletion, immune activation, and dysfunction in NRG-hu HSC and NRG-hu Thy/HSC mice. Moreover, combined antiretroviral therapy (cART) inhibited HIV-1 replication efficiently with similar persistent HIV-1 reservoirs in both models. Finally, we found that blocking type-I interferon signaling under cART treatment transiently activated HIV-1 reservoirs, enhanced T cell recovery and reduced HIV-1 reservoirs in both HIV-1 infected NRG-hu HSC and NRG-hu Thy/HSC mice. In summary, we report that NRG-hu Thy/HSC and NRG-hu HSC mice support similar HIV-1 infection and similar HIV-1 immunopathology; and HIV-1 replication responds similarly to cART and IFNAR blockade therapies. The NRG-hu HSC mouse model reconstituted with human HSC only is sufficient for the study of HIV-1 infection, pathogenesis, and therapy.

## Introduction

Human immunodeficiency virus type 1 (HIV-1) infects and progressively depletes CD4^+^ T cells, causing acquired immune deficiency syndrome (AIDS). Approximately 70 million people have been infected with HIV-1, and half of them have died of HIV/AIDS-related causes (1). The development of combined antiretroviral therapy (cART), which can efficiently suppress viral replication, has significantly improved survival and life quality of HIV-1-infected patients who can both access and tolerate cART (2). However, cART is not curative and must be continued for life (3, 4). Moreover, lifelong treatment is associated with significant side effects and non-AIDS-related “end-organ disease” (5). Thus, there is a great need for the development of novel therapies that can both control the epidemic and cure those individuals who have already been infected with HIV-1.

Understanding how HIV-1 infection leads to immunodeficiency is key for the development of new treatments. After more than 30 years of research, the precise mechanism by which HIV-1 infection causes AIDS development is still poorly understood, mainly due to the lack of robust small animal models. The recent development of humanized mice with functional human immune systems offer a relevant and robust model for the study of HIV-1 infection, replication, pathogenesis, and therapies (6–8). Humanized mice were constructed by transplantation of human CD34^+^ hematopoietic stem cells and/or implantation of human thymus tissue into immunodeficient mice, such as the NOD-scid γ_c_^−/−^ (NSG) mice and NOD-Rag2^−/−^γ_c_^−/−^ (NRG) mice (9). Two major humanized mouse models, the NRG-hu HSC model and the hu-BLT model, are widely used for HIV-1 studies. The NRG-hu HSC model involves preconditioning neonate immunodeficient mice with radiation and then injecting them with human CD34^+^ HSCs (10–13). In the hu-BLT model, implantation of human thymus tissue under the kidney capsule is combined with HSCs infusion into irradiated adult immunodeficient mice (14, 15). We and others have reported that all the major human lymphoid lineage including T cell, B cell, and innate immune cells including NK cell, monocytes, myeloid dendritic cells (mDC), and plasmacytoid dendritic cells (pDC) are developed in both NRG-hu HSC mice and hu-BLT mice (10–20).

Both NRG-hu HSC and hu-BLT models can develop significant levels of innate and adaptive immune responses (21–25) and can be infected by HIV-1 (7, 26–28). HIV-1 infection leads to progressive CD4^+^ T cell depletion in both peripheral blood and lymphoid tissues (8). Moreover, like in humans, HIV-1 infection also leads to T cell activation and exhaustion in both NRG-hu HSC and hu-BLT mice (29–31). HIV-1 infection can be treated with the antiretroviral drugs that are used in infected humans (32–36). Also like in humans, antiretroviral treatment of HIV-1 infection results in systemic recovery of CD4 T cells in humanized mice. In addition, both mouse models are used for testing the effectiveness of immunotherapy to inhibit HIV-1 replication, reverse HIV-1 induced immunopathology and control HIV-1 reservoir (25, 29, 30, 34, 37–39).

The advantage of the NRG-hu HSC model is that the procedure to construct the mice is simple, only involving pre-irradiating the neonate immunodeficient mice followed by injecting human CD34^+^ HSCs (10, 11, 13). To generate hu-BLT mice, a time consuming and technically difficult surgery procedure is needed to implant the human thymus tissue into the kidney capsule of the mice (14, 15). Another major difference between these two models is that in NRG-hu HSC mouse, the human T cells are produced in the mouse thymus and presumed to be educated in the context of mouse major histocompatibility complex (MHC) (10–13). In hu-BLT mice, human T cells can develop in the presence of human thymic epithelium, resulting in human MCH-restricted T cells (14, 15, 40). Thus, although both models are versatile tools for HIV-1 study, parallelly study to compare the human immune reconstitution, HIV-1 replication, immunopathology, and responses to therapy in both models will help to guide researchers how to balance and decide which system to use. In this study, we performed a comprehensive parallel comparison of human immune reconstitution and HIV-1 replication, immunopathogenesis and therapy between newborn immunodeficient mice transplanted with HSCs (NRG-hu HSC) and 6- to 8-week-old adult mice transplanted with HSCs and thymus (NRG-hu Thy/HSC) from same human donors into same background of immunodeficient mice. We report that both NRG-hu HSC and NRG-hu Thy/HSC mice support significant levels of human immune reconstitution and comparable levels of HIV-1 replication, immunopathology, and responses to cART and immune therapy.

## Materials and Methods

### Construction of Humanized Mice

NRG (NOD-Rag2^−/−^γc^−/−^) mice were obtained from the Jackson Laboratory. Human fetal liver and thymus (gestational age of 16–20 weeks) were obtained from medically or elective indicated termination of pregnancy through a non-profit intermediary working with outpatient clinics (Advanced Bioscience Resources, Alameda, CA, USA). Written informed consent of the maternal donors is obtained in all cases, under regulation governing the clinic. NRG-hu HSC mice were generated by intrahepatic injection of new born (1–5 days old) NRG mice (irradiated at 200 cGy from a 137Cs gamma radiation source) with 3 × 10^5^ human fetal liver derived CD34^+^ HSCs as previously reported (41). To generate NRG-hu Thy/HSC mice, 6- to 8-week-old NRG mice were sub-lethally irradiated (250 cGy) and anesthetized, and ~1-mm^3^ fragments of human fetal thymus fragments were implanted under the kidney capsule. 5 × 10^5^ CD34^+^ HSCs purified from fetal liver of the same donor were injected i.v. within 3 h. All mice were housed and bred in a specific pathogen-free environment. All animal studies were approved by the University of North Carolina Institutional Animal Care and Use Committee.

### Antibodies and Flow Cytometry

Antibodies to CD45 (HI30), CD4 (RPA-T4), CD8 (HIT8a), CD56 (5.1h11), CD123 (6H6), CD14 (63D3), CD11c (3.9), CD45RA (HI100), CCR7 (G043H7), CD10 (HI10a), IL-2 (MQ1-17H12), IFN-γ (4S.B3), HLA-DR (L243), CD38 (HIT2), and PD-1 (EH12.2H7) were purchased from BioLegend. Antibodies to CD3 (7D6), CD19 (6D5), mouse CD45 (30-F11), and LIVE/DEAD Fixable Yellow Dead Cell Stain Kit were purchased from Invitrogen. Antibody to HIV-1 p24 (KC57) were purchased from Beckman Coulter.

Total lymphocytes were isolated prepared from peripheral blood, spleen, bone marrow (BM), and mesenteric lymph nodes (mLNs) according to standard protocols; red blood cells were lysed with ACK buffer. Intrahepatic lymphocytes were prepared as described (42). Total cell number was quantified by Guava Easycytes with Guava Express software (Guava). For surface staining, single cell suspension was stained with surface markers and analyzed on a CyAn ADP flow cytometer (Dako). For intracellular staining, cells were first stained with surface markers and then fixed and permeabilized with cytofix/cytoperm buffer (BD Bioscience), followed by intracellular staining. Data were analyzed using Summit4.3 software (Dako).

### T Cell Stimulation and Intracellular Cytokine Staining

Splenocytes from humanized mice were stimulated *ex vivo* with PMA (phorbol 12-myristate 13-acetate) (50 ng/ml) and ionomycin (1 μM) (Sigma, St. Louis, MO, USA) for 4 h in the presence of brefeldin A (BioLegend). Cell were then fixed and permeabilized with cytofix/cytoperm buffer (BD Biosciences), and intracellular staining was then performed.

### TLR-L Treatment *In Vivo*

CpG-B (ODN 2006), R848, and poly I:C were all purchased from InvivoGen. For *in vivo* treatment, humanized mice were treated with 50 μg/mouse of CpG-B, poly I:C or 20 μg/mouse R848 through i.p. injection.

### Detection of Cytokines in Plasma

Human pan IFN-α (subtypes 1/13, 2, 4, 5, 6, 7, 8, 10, 14, 16, and 17) were detected by enzyme-linked immunosorbent assay using the human IFN-α pan ELISA kits purchased from Mabtech (Nacka Strand, Sweden). Human IL-6 in plasma of humanized mice were detected by immunology multiplex assay (Luminex) (Millipore, Billerica, MA, USA).

### HIV-1 Infection of Humanized Mice

The CCR5-tropic strain of HIV-1 (JR-CSF) was generated by transfection of 293T cells (ATCC) with plasmid containing full length HIV-1 (JR-CSF) genome. Humanized mice with stable human leukocyte reconstitution were anesthetized and infected with HIV-1 (JR-CSF) (10 ng p24/mouse) through retro-orbital injection.

### HIV-1 Genomic RNA Detection in Plasma

HIV-1 RNA was purified from the plasma with the QIAamp^®^ Viral RNA Mini Kit. The RNA was then reverse transcribed and quantitatively detected by real-time PCR using the TaqMan^®^ Fast Virus 1-Step PCR kit (ThermoFisher Scientific). The primers used for detecting the HIV Gag gene were (5′-GGTGCGAGAGCGTCAGTATTAAG-3′ and 5′-AGCTCCCTGCTTGCCCATA-3′). The probe (FAM-AAAATTCGGTTAAGGCCAGGGGGAAAGAA-QSY7) used for detection was ordered from Applied Biosystems, and the reactions were set up following the manufacturer’s guidelines and were run on the QuantStudio 6 Flex PCR system (Applied Biosystems). The detection limit of the real-time PCR reaction is four copies per reaction. Accordingly, the limit of detection of the assay with 50 µl of blood is 400 copies/ml in humanized mice.

### Combination Antiretroviral Therapy

Food formulated with antiretroviral individual drug was prepared as reported with elevated dose modifications (34). In brief, tablets of emtricitabine and tenofovir disoproxil fumarate (Truvada^®^; Gilead Sciences) and raltegravir (Isentress^®^; Merck) were crushed into fine powder and manufactured with TestDiet 5B1Q feed (Modified LabDiet 5058 with 0.12% amoxicillin) into 1/2” irradiated pellets. Final concentrations of drugs in the food were 4,800 mg/kg raltegravir, 1,560 mg/kg tenofovir disoproxil, and 1,040 mg/kg emtricitabine. The estimated drug daily doses were 768 mg/kg raltegravir, 250 mg/kg tenofovir disoproxil, and 166 mg/kg emtricitabine.

### *In Vivo* IFNAR1 Blocking Antibody Treatments

The α-IFNAR1 monoclonal antibody (mAb) was generated as previous reported (29). To block type-I interferon (IFN-I) signaling during chronic HIV-1 infection, humanized mice were treated i.p. with IFNAR1 blocking antibodies twice a week with the dose 400 µg/mouse at the first injection and 200 μg/mouse for the following treatments. Cohorts of mice were randomized into different treatment groups by level of HIV-1 RNA in plasma.

### Cell-Associated HIV-1 DNA Detection

To measure total cell-associated HIV-1 DNA, nucleic acid was extracted from spleen and BM cells using the DNeasy Blood & Tissue Kit (Qiagen). HIV-1 DNA was quantified by real-time PCR. DNA from serial dilutions of ACH2 cells, which contain one copy of HIV genome in each cell, was used to generate a standard curve.

### Viral Outgrowth Assay

Viral outgrowth assay was performed as reported (43). Serial dilutions of human cells from splenocytes of humanized mice (1 × 10^6^, 2 × 10^5^, and 4 × 10^4^ human cells) were stimulated with PHA (2 µg/ml) and IL-2 (100 U/ml) for 24 h. MOLT4/CCR5 cells were added on day 2 to enhance the survival of the cultured cells as well as to support and facilitate further HIV-1 replication. Culture medium containing IL-2 (NIH AIDS reagents program) and T cell growth factor (homemade as describe in the standard protocol) was replaced on days 5 and 9. After 7 and 14 days of culture, supernatant from each well was harvested, and HIV-1 RT-qPCR was performed to score viral outgrowth. Estimated frequencies of cells with replication-competent HIV-1 were determined by maximum likelihood statistics (43).

### Statistical Analysis

In all other experiments, significance levels of data were determined by using Prism5 (GraphPad Software). Experiments were analyzed by two-tailed Student’s *t*-test, or by one-way analysis of variance (ANOVA) and Bonferroni’s *post hoc* test according to the assumptions of the test, as indicated for each experiment. A *P* value less than 0.05 was considered significant. The number of animals and replicates is specified in each figure legend.

## Results

### Human Lymphoid and Myeloid Lineage Cells Are Reconstituted in Peripheral Blood of Both NRG-hu HSC and NRG-hu Thy/HSC Mice

**Figure 1:**
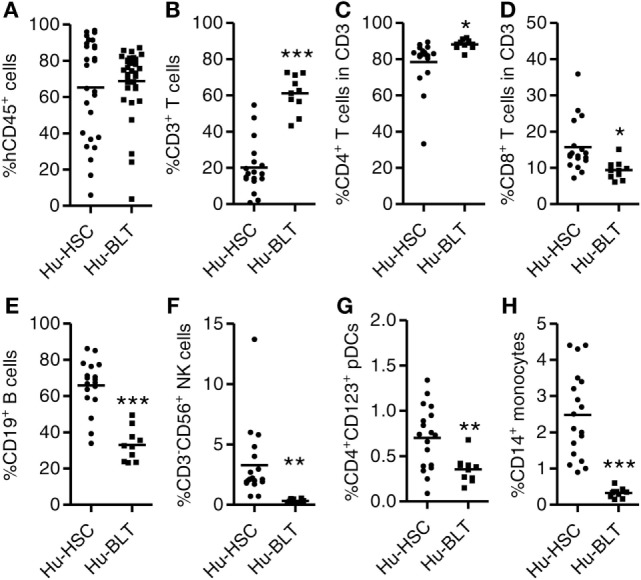
Comparison of human immune cell reconstitution in peripheral blood of NRG-hu HSC and NRG-hu Thy/HSC mice. NRG-hu HSC and NRG-hu Thy/HSC mice were generated as indicated in Section “Materials and Methods.” **(A)** Summarized data show percentage of human leukocytes (hCD45^+^) in total peripheral blood leukocytes 12 weeks after reconstitution. Shown are combined data from two cohorts of NRG-hu HSC mice (*n* = 27) and NRG-hu Thy/HSC mice (*n* = 35) reconstituted with HSCs ± thymus from the same human donor. **(B–H)** Summarized data show percentage of human T cells (CD3^+^), B cells (CD19^+^), NK cells (CD3^−^CD56^+^), plasmacytoid dendritic cells (pDCs) (CD3^−^CD19^−^HLA-DR^+^CD4^+^CD123^+^), and monocytes (CD3^−^CD19^−^HLA-DR^+^CD14^+^) in human CD45^+^ cells **(B,E–H)** and percentage of human CD4 T and CD8 T cells in human CD3 T cells **(C,D)**. Shown are representative data **(B–H)** from *n* = 18 (NRG-hu HSC) and *n* = 10 (NRG-hu Thy/HSC) hu-mice reconstituted with HSCs ± thymus from same donor. Each dot represents one individual mouse; bars indicate mean (*P < 0.05, **P < 0.01, and ***P < 0.001, by unpaired, two-tailed Student’s *t*-test).

To compare the level of human immune reconstitution in humanized mice (hu-mice) transplanted with human HSCs only (NRG-hu HSC) or with human HSCs plus thymus tissue (NRG-hu Thy/HSC), we reconstituted newborn NRG mice with human fetal liver derived CD34^+^ HSCs (NRG-hu HSC) or reconstituted 6- to 8-week-old NRG mice with human fetal liver derived CD34^+^ HSCs together with fetal thymus tissue (NRG-hu Thy/HSC) from the same donor. The difference between the NRG-hu Thy/HSC model and the hu-BLT model is that we only transplant thymus tissue but not fetal liver tissue under the kidney capsule. The other difference is that we transplant human HSCs within 3 h after human thymus transplantation. As reported in hu-BLT mice (14, 15), human thymic organoid was well developed and showed long-term sustained thymopoiesis in NRG-hu Thy/HSC mice (Figure S1 in Supplementary Material). Human immune cell reconstitution in the peripheral blood was detected by flow cytometry 12 weeks after transplantation. All major human CD45^+^ leukocyte subsets including T cells (CD3^+^), B cells (CD19^+^), NK cells (CD3^−^CD56^+^), monocytes (CD3^−^CD19^−^HLA^−^DR^+^CD14^+^), and pDCs (CD3^−^CD19^−^HLA^−^DR^+^CD4^+^CD123^+^) were detected in peripheral blood of both NRG-hu HSC and NRG-hu Thy/HSC mice (Figure 1; Figure S1 in Supplementary Material). Similar level of human CD45^+^ cells was found in NRG-hu HSC (65.3 ± 5.3%) and NRG-hu Thy/HSC (68.8 ± 3.1%) mice (Figure 1A). The percentage of human T cells within human CD45^+^ leukocytes was significantly higher in NRG-hu Thy/HSC mice (61.1 ± 3.2%) compared with the level in NRG-hu HSC mice (20.1 ± 3.4%) (Figure 1B). The result indicated that fetal liver/thymus “sandwich” structure (14, 15) is not essential for the long-term functioning human thymus development if human CD34^+^ HSCs were transplanted immediately after thymus transplantation. Progenitor cells derived from CD34^+^ HSCs can serve as the source of thymocyte progenitors. Both CD4 and CD8 T cells were developed in NRG-hu HSC and NRG-hu Thy/HSC mice (Figures 1C,D). The ratio of CD4 T cells to CD8 T cells was slightly higher in NRG-hu Thy/HSC mice compare to NRG-hu HSC mice (Figures 1C,D). The percentage of human B cells was 65.8 ± 3.3% in NRG-hu HSC mice and 33 ± 4.9% in NRG-hu Thy/HSC mice (Figure 1E). The percentages of human NK cells, pDCs, and monocytes were also lower in NRG-hu Thy/HSC mice compared with NRG-hu HSC mice (Figures 1F–H).

### Human Leukocytes Are Equally Reconstituted in Lymphoid Organs in Both NRG-hu HSC and NRG-hu Thy/HSC Mice

**Figure 2:**
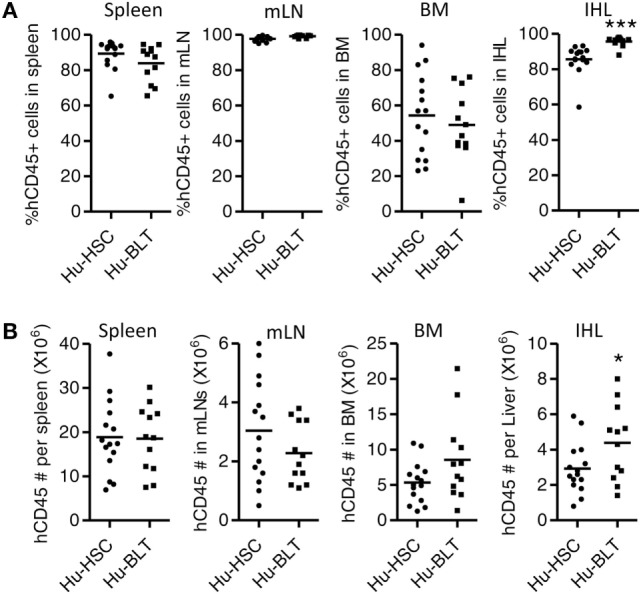
Comparison of total human immune cell reconstitution in lymphoid organ of NRG-hu HSC and NRG-hu Thy/HSC mice. NRG-hu HSC and NRG-hu Thy/HSC mice were terminated at 20 weeks after reconstitution and human immune cells in lymphoid organs were detected by flow cytometry. Summarized data show percentage **(A)** and number **(B)** of human leukocytes (hCD45^+^) in spleen, mesenteric lymph nodes (mLNs), bone marrow (BM), and intrahepatic leukocytes (IHL) of NRG-hu HSC and NRG-hu Thy/HSC mice. Shown are representative data from *n* = 15 (NRG-hu HSC) and *n* = 12 (NRG-hu Thy/HSC) hu-mice per group reconstituted with HSCs ± thymus from same donor. Each dot represents one individual mouse; bars indicate mean (*P < 0.05 and ***P < 0.001, by unpaired, two-tailed Student’s *t*-test).

We also detected human immune reconstitution in lymphoid organs including spleen, mLNs, liver, and BM of NRG-hu HSC and NRG-hu Thy/HSC mice. The percentage of human CD45^+^ cell and total number of human CD45^+^ cells were comparable in spleen, mLN, and BM between NRG-hu HSC and NRG-hu Thy/HSC mice (Figures 2A,B). The level of human immune cells in the liver was slightly higher in NRG-hu Thy/HSC mice (95.7 ± 0.8%) compared with the NRG-hu HSC mice (85.6 ± 2.2%), consistent with the higher number of human CD45^+^ cells in the liver in NRG-hu Thy/HSC mice (Figures 2A,B).

### Similar Phenotype and Function of Human T and B Cells Developed in NRG-hu HSC and NRG-hu Thy/HSC Mice

**Figure 3:**
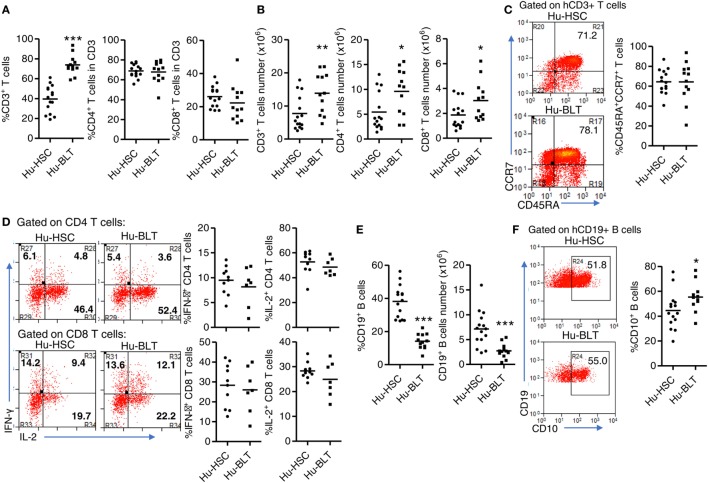
Phenotype and function of human T and B cells from NRG-hu HSC and NRG-hu Thy/HSC mice. NRG-hu HSC and NRG-hu Thy/HSC mice were terminated at 20 weeks after reconstitution. **(A,B)** Summarized data show percentage **(A)** and number **(B)** of total human T cells, CD4 T and CD8 T cells in spleen of NRG-hu HSC and NRG-hu Thy/HSC mice. **(C)** Representative dot plots and summarized data show the expression of CD45RA and CCR7 on CD3 T cells from the spleen of NRG-hu HSC and NRG-hu Thy/HSC mice detected by flow cytometry. Shown **(A–C)** are data from *n* = 15 (NRG-hu HSC) and *n* = 12 (NRG-hu Thy/HSC) hu-mice per group reconstituted with HSCs/thymus from same donor. **(D)** Splenocytes were stimulated *ex vivo* with PMA/ionomycin for 4 h followed by intracellular cytokine staining. Representative dot plots and summarized data show percentages of IFN-γ and IL-2 producing CD4 and CD8 T cells. Shown are data from *n* = 10 (NRG-hu HSC) and *n* = 7 (NRG-hu Thy/HSC) hu-mice per group. **(E)** Summarized data showed percentage and number of total human B cells in spleen of NRG-hu HSC and NRG-hu Thy/HSC mice. **(F)** Representative dot plots and summarized data show expression of CD10 on B cells from the spleen of NRG-hu HSC and NRG-hu Thy/HSC mice. Shown **(E,F)** are data from *n* = 15 (NRG-hu HSC) and *n* = 12 (NRG-hu Thy/HSC) hu-mice per group reconstituted with HSCs ± thymus from the same donor. Each dot represents one individual mouse; bars indicate mean (*P < 0.05, **P < 0.01, and ***P < 0.001, by unpaired, two-tailed Student’s *t*-test).

We next determined the phenotype and function of human T cells and B cells from spleen of NRG-hu HSC and NRG-hu Thy/HSC mice. As in the peripheral blood, both the percentage and number of CD3^+^ T cells were higher in the spleen of NRG-hu Thy/HSC mice (Figures 3A,B). The percentages of CD4 and CD8 T cells in total T cells did not show difference in the spleen between these two models (Figure 3A). Most of the T cells from both NRG-hu HSC (64.4 ± 3.1%) and NRG-hu Thy/HSC (64.3 ± 5.8%) showed naïve phenotype at 20 weeks posttransplantation (Figure 3C). The function of T cells from both humanized mouse models were equal as they produced similar level of IFN-γ and IL-2 in response to mitogen stimulation *ex vivo* (Figure 3D). No spontaneous IFN-γ and IL-2 production by T cells was detected from either the NRG-hu HSC or NRG-hu Thy/HSC mice.

As in the peripheral blood, the percentage and number of B cells in the spleen were lower in NRG-hu Thy/HSC mice compared with the NRG-hu HSC mice (Figure 3E). It has been reported that human B cells developed in humanized mice were immature and cannot produce significant level of antigen-specific IgG by vaccination (44–46). We also compared the phenotype of B cells from both NRG-hu HSC and NRG-hu Thy/HSC mice and found that they both express high level of immature marker CD10 (Figure 3F). The expression of CD10 on B cells from NRG-hu Thy/HSC mice was slightly higher (Figure 3F), indicating that co-transplantation of thymus had minor effect on the maturation of B cells in NRG-hu Thy/HSC mice.

### Innate Immune Cells Were Developed in Spleen of Both NRG-hu HSC and NRG-hu Thy/HSC Mice and Responded Similarly to TLR-Ls Stimulation

**Figure 4:**
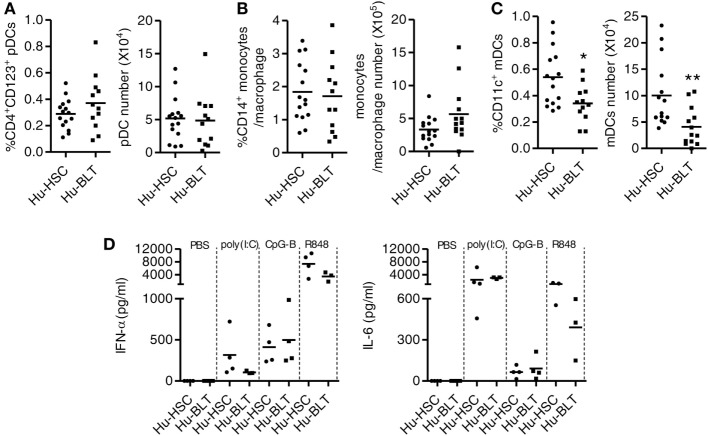
Development and function of human innate immune cells from NRG-hu HSC and NRG-hu Thy/HSC mice. NRG-hu HSC and NRG-hu Thy/HSC mice were terminated at 20 weeks after reconstitution. **(A–C)** Summarized data show percentage and number of total human plasmacytoid dendritic cells (pDCs) (CD3^−^CD19^−^HLA-DR^+^CD4^+^CD123^+^), monocytes or macrophage (CD3^−^CD19^−^HLA-DR^+^CD14^+^), and myeloid dendritic cell (mDC) (CD3^−^CD19^−^HLA-DR^+^CD11c^+^CD14^−^) in spleen. Shown **(A–C)** are data from *n* = 15 (NRG-hu HSC) and *n* = 12 (NRG-hu Thy/HSC) hu-mice per group reconstituted with HSCs ± thymus from same donor. **(D)** NRG-hu HSC and NRG-hu Thy/HSC mice were injected intraperitoneally with CpG-B (50 μg/mouse), poly I:C (50 μg/mouse), R848 20 μg/mouse, or PBS. Plasma was collected at different time points posttreatment. IFN-α levels in plasma were detected at 24 h after treatment. IL-6 level was detected at 4 h after treatment. Shown **(D)** are data from three to four mice each group for each treatment conditions. Each dot represents one individual mouse; bars indicate mean (*P < 0.05 and **P < 0.01, by unpaired, two-tailed Student’s *t*-test).

We compared the reconstitution of human innate immune cells including pDCs, mDCs and monocytes/macrophages in the spleen of NRG-hu HSC and NRG-hu Thy/HSC mice. The percentage and number of pDC (Figure 4A) and monocytes/macrophage (Figure 4B) were comparable, while there were more mDCs in the spleen of NRG-hu HSC mice compared with NRG-hu Thy/HSC mice (Figure 4C).

To detect the function of innate immune cells developed in NRG-hu HSC and NRG-hu Thy/HSC mice, we treated the mice *in vivo* with the TLR9-ligands CpG-B, the TLR7/8-L R848 and the TLR3-L poly I:C and detected cytokine production in the serum. We found that all the three TLR-Ls induced significant levels of IFN-α and IL-6 in both NRG-hu HSC and NRG-hu Thy/HSC mice (Figure 4D). The induction of IFN-α by poly(I:C) and R848 stimulation is slightly lower in NRG-hu Thy/HSC mice compared with NRG-hu HSC mice (Figure 4D) which probably due to the lower number of mDC developed in NRG-hu Thy/HSC mice compared with NRG-hu HSC mice (Figure 4C).

In summary, most human lymphoid and myeloid lineage cells are reconstituted in both NRG-hu HSC and NRG-hu Thy/HSC mice, with human T cells predominantly developed in NRG-hu Thy/HSC mice, while NRG-hu HSC mice support better human B cell and myeloid cell development. The phenotype and function of human immune cells developed in NRG-hu HSC mice and NRG-hu Thy/HSC mice are similar.

### NRG-hu HSC and NRG-hu Thy/HSC Mice Support Similar Level of HIV-1 Replication *In Vivo*

**Figure 5:**
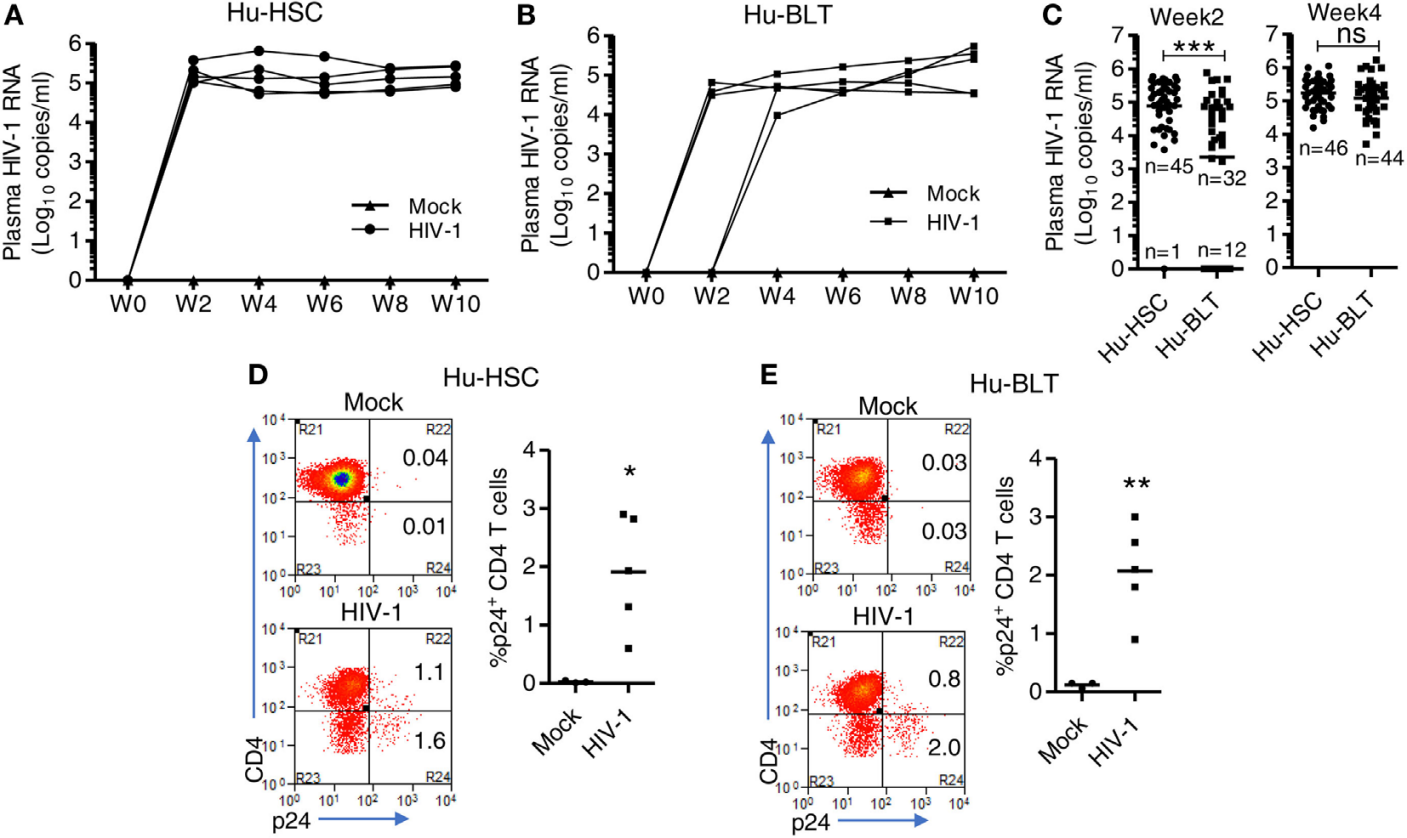
HIV-1 replication kinetics in NRG-hu HSC and NRG-hu Thy/HSC mice *in vivo*. NRG-hu HSC and NRG-hu Thy/HSC mice were infected with HIV-1 at 12–13 weeks postinfection (wpi). **(A,B)** Plasma HIV-1 genomic RNA levels were measured at indicated time points in each NRG-hu HSC **(A)** and NRG-hu Thy/HSC **(B)** mice. Shown are representative data from *n* = 3 (NRG-hu HSC/Mock), *n* = 5 (NRG-hu HSC/HIV-1), *n* = 3 (NRG-hu Thy/HSC/Mock), and *n* = 5 (NRG-hu Thy/HSC/HIV-1) mice transplanted with HSCs ± thymus from the same donor. The broken horizontal line indicates the limit of detection (400 copies/ml). **(C)** Summary data show plasma HIV-1 genomic RNA levels at 2 and 4 wpi from NRG-hu HSC (*n* = 46) and NRG-hu Thy/HSC (*n* = 44) mice. Shown **(C)** are combined data from five cohorts of NRG-hu HSC mice and six cohorts of NRG-hu Thy/HSC mice. The viral infection data were collected previously by the laboratory. **(D,E)** Representative FACS plots and summarized data show percentages of HIV-1 p24-positive CD4 T cells (CD3^+^CD8^−^) in the spleen of NRG-hu HSC **(D)** and NRG-hu Thy/HSC **(E)** mice at 10 wpi. Shown are representative data from *n* = 3 (NRG-hu HSC/Mock), *n* = 5 (NRG-hu HSC/HIV-1), *n* = 3 (NRG-hu Thy/HSC/Mock), and *n* = 5 (NRG-hu Thy/HSC/HIV-1) mice transplanted with HSCs ± thymus from the same donor. Each dot represents one individual mouse; bars indicate mean (*P < 0.05, **P < 0.01, and ***P < 0.001, by unpaired, two-tailed Student’s *t*-test).

We and others have reported that both NRG-hu HSC and NRG-hu Thy/HSC mice supported HIV-1 replication *in vivo* (7, 8, 47). Here we compared the HIV-1 replication kinetics in NRG-hu HSC mice and NRG-hu Thy/HSC mice transplanted with HSCs ± thymus from the same donor tissue. We found the viremia reached to peak level at 2 weeks postinfection (wpi) in NRG-hu HSC mice (Figure 5A), while in NRG-hu Thy/HSC mice, the viremia reached the peak level at 4 wpi (Figure 5B). At 2 wpi, NRG-hu HSC mice supported efficient HIV-1 replication in nearly all mice (98%) but NRG-hu Thy/HSC mice supported HIV-1 replication in about 73% of infected mice (Figure 5B). The results suggest that the immune cells in NRG-hu Thy/HSC mice may control/delay HIV-1 replication at the early stage of HIV-1 infection. At 4 wpi, all the infected BLT mice showed similar viremia as detected in NRG-hu HSC mice and sustained through 10 wpi when we terminated the mice (Figures 5A–C). HIV-1 p24 levels in CD4 T cells from the spleen were similar in NRG-hu HSC and NRG-hu Thy/HSC mice at 10 wpi (Figures 5D,E). In summary, the results indicated that both the NRG-hu HSC and NRG-hu Thy/HSC mice support similar levels of HIV-1 replication, although there was a 2 weeks delay reaching the peak viremia in NRG-hu Thy/HSC mice.

### HIV-1 Infection Induces Similar Levels of T Cell Depletion, Activation, and Exhaustion in NRG-hu HSC and NRG-hu Thy/HSC Mice

**Figure 6:**
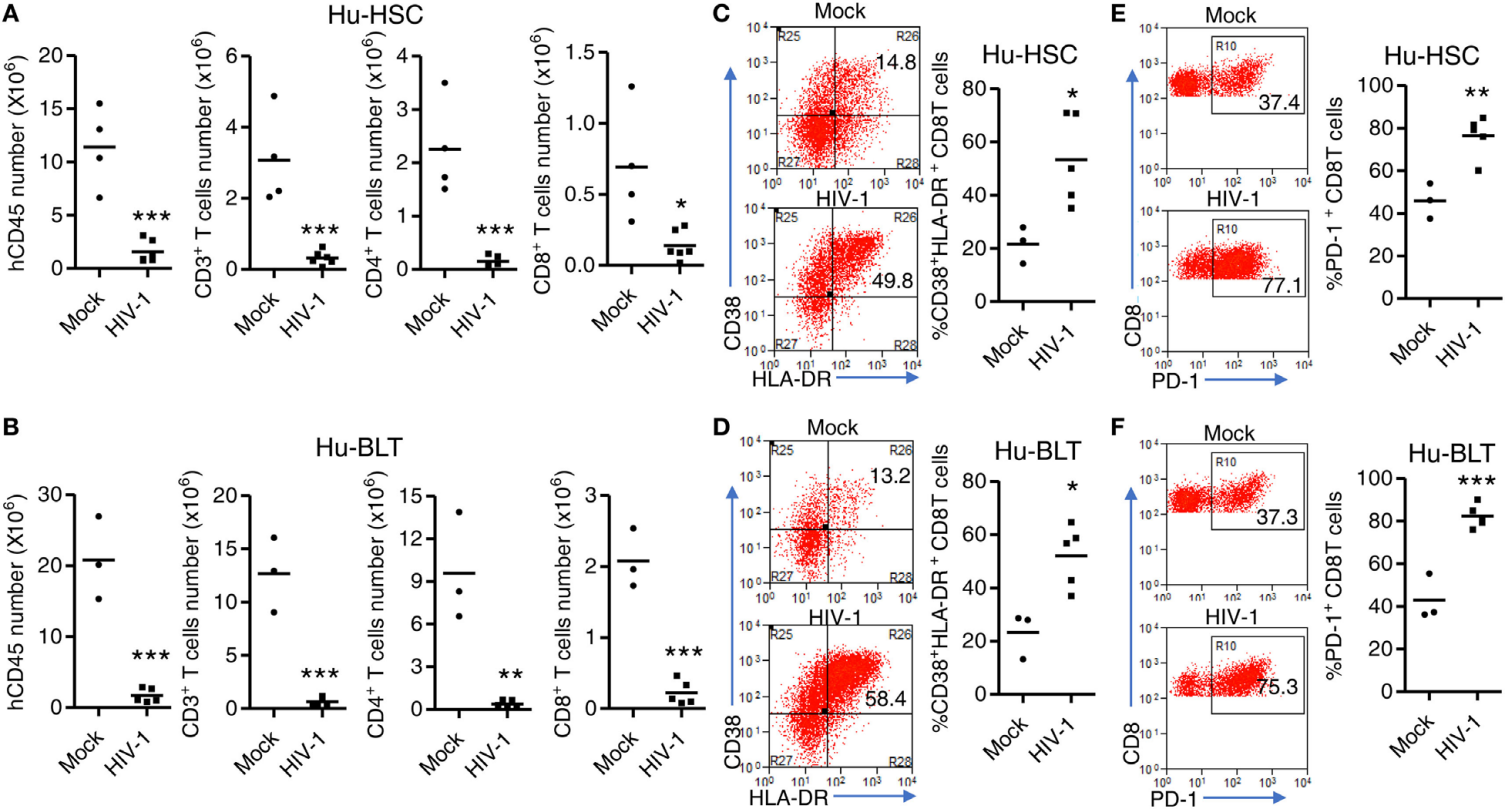
HIV-1-induced immunopathology in NRG-hu HSC and NRG-hu Thy/HSC mice. NRG-hu HSC and NRG-hu Thy/HSC mice were infected with HIV-1. Mice were sacrificed at 10 weeks postinfection. **(A,B)** Numbers of total human leukocytes, CD3^+^ T cells, CD4 T cells (CD3^+^CD8^−^), and CD8 T cells (CD3^+^CD8^−^) and in spleens of NRG-hu HSC **(A)** and NRG-hu Thy/HSC **(B)** mice. **(C,D)** Representative FACS plots and summarized data show the expression of CD38 and HLA-DR on CD8 T cells from spleen of NRG-hu HSC **(C)** and NRG-hu Thy/HSC **(D)** mice. **(E,F)** Representative FACS plots and summarized data show the expression of PD-1 on CD8 T cells from spleen of NRG-hu HSC **(E)** and NRG-hu Thy/HSC **(F)** mice. Shown are representative data from *n* = 3 (NRG-hu HSC/Mock), *n* = 5 (NRG-hu HSC/HIV-1), *n* = 3 (NRG-hu Thy/HSC/Mock), and *n* = 5 (NRG-hu Thy/HSC/HIV-1) mice reconstituted with HSCs/thymus from the same donor. Each dot represents one individual mouse; bars indicate mean (*P < 0.05, **P < 0.01, and ***P < 0.001, by unpaired, two-tailed Student’s *t*-test).

We next detected HIV-1 induced immunopathology including T cell depletion, activation and dysfunction in both NRG-hu HSC and NRG-hu Thy/HSC mice. We found that HIV-1 induced similar level of total human CD45^+^ cells and human T cells depletion in both NRG-hu HSC and NRG-hu Thy/HSC mice (Figures 6A,B). HIV-1 infection also induced similar level of CD38 and HLA-DR expression on CD8 T cells in both NRG-hu HSC and NRG-hu Thy/HSC mice (Figures 6C,D). In addition, we detected the T cells exhaustion marker PD-1 expression and found that both CD8 T cell from NRG-hu HSC and NRG-hu Thy/HSC mice expressed higher level of PD-1 than mock-infected mice, which indicated HIV-1 induced human T cell exhaustion in both humanized mouse models (Figures 6E,F). The results indicate that HIV-1 infection induced T cell depletion, activation and exhaustion to similar levels in both NRG-hu HSC and NRG-hu Thy/HSC mice.

### cART Efficiently Inhibits HIV-1 Replication in Both NRG-hu HSC and NRG-hu Thy/HSC Mice

**Figure 7:**
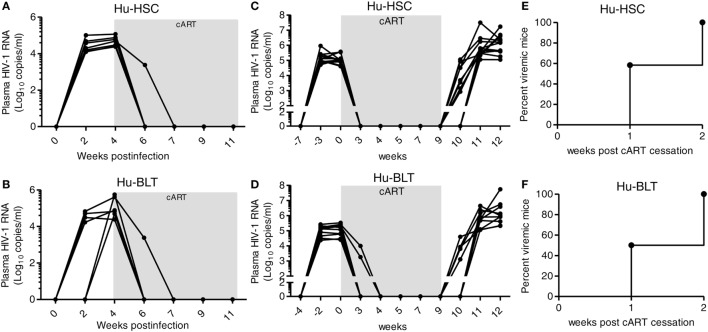
Similar efficacy of combined antiretroviral therapy (cART) response in HIV-1-infected NRG-hu HSC and NRG-hu Thy/HSC mice. **(A,B)** NRG-hu HSC (*n* = 6) and NRG-hu Thy/HSC mice (*n* = 7) reconstituted with HSCs ± thymus from the same donor were infected with HIV-1 and treated with cART from 4 to 11 weeks postinfection. HIV-1 genomic RNA levels in the plasma were detected at indicated time points. **(C,D)** A different cohort of NRG-hu HSC (*n* = 12) and NRG-hu Thy/HSC mice (*n* = 10) were infected with HIV-1 and treated with cART for 9 weeks. HIV-1 genomic RNA levels in the plasma were detected before and after cART discontinuation at indicated time points. The broken horizontal line indicates the limit of detection (400 copies/ml). **(E,F)** Kinetic analysis of HIV-1 rebound post-cART cessation. Shown **(C–E)** are combined data from *n* = 12 (NRG-hu HSC) and *n* = 10 (NRG-hu Thy/HSC) mice.

We and others have shown before that as in human patients, cART can efficiently inhibit HIV-1 replication in hu-mice (29, 33, 34, 38). We compared the efficacy of cART to inhibit HIV-1 replication in NRG-hu HSC mice and NRG-hu Thy/HSC mice. The results indicate that plasma viremia decreased to undetectable levels (<400 genome copies/ml) in all HIV-infected NRG-hu HSC and NRG-hu Thy/HSC mice within 3 weeks after cART treatment (Figures 7A,B). Similar to cART-treated patients, HIV-1 reservoirs persisted stably in both NRG-hu HSC and NRG-hu Thy/HSC mice and virus rebounded rapidly after cART cessation (Figures 7C,D). HIV-1 rebounded in 60% NRG-hu HSC mice at 1-week post cART cessation and in 100% NRG-hu HSC mice at 2 weeks post cART cessation (Figure 7E). Similarly, HIV-1 rebounded in 50% NRG-hu Thy/HSC mice at 1-week post cART cessation and in 100% NRG-hu Thy/HSC mice at 2 weeks post cART cessation (Figure 7F).

### IFNAR Blockade During cART-Suppressed HIV-1 Infection Reverses Aberrant Immune Activation and Exhaustion Phenotype of Human T Cells

**Figure 8:**
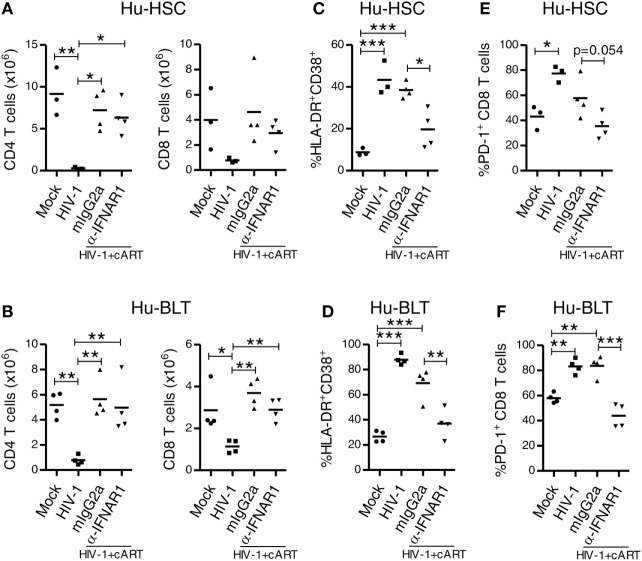
IFNAR blockade reduces activation and PD-1 expression on CD8 T cells in both HIV-1 infected NRG-hu HSC and NRG-hu Thy/HSC mice under combined antiretroviral therapy (cART). NRG-hu HSC and NRG-hu Thy/HSC mice infected with HIV-1 were treated with cART from 4 to 12 weeks postinfection (wpi). From 7 to 10 wpi, the cART-treated mice were injected with α-IFNAR1 antibody or isotype control mIgG2a antibody. Mice were sacrificed at 12 wpi. **(A,B)** Summarized data show numbers of human CD4 and CD8 T cells from spleens of NRG-hu HSC **(A)** and NRG-hu Thy/HSC **(B)** mice. **(C,D)** Summarized data show percent HLA-DR^+^CD38^+^ of CD8 T cells from spleens of NRG-hu HSC **(C)** and NRG-hu Thy/HSC **(D)** mice. **(E,F)** Summarized data show percent PD-1^+^ of CD8 T cells from spleens of NRG-hu HSC **(E)** and NRG-hu Thy/HSC **(F)** mice. Shown are representative data from *n* = 3 (NRG-hu HSC/Mock), *n* = 3 (NRG-hu HSC/HIV-1), *n* = 4 (NRG-hu HSC/HIV-1/cART/mIgG2a), *n* = 4 (NRG-hu HSC/HIV-1/cART/α-IFNAR1), *n* = 4 (NRG-hu Thy/HSC/Mock), *n* = 4 (NRG-hu Thy/HSC/HIV-1), and *n* = 4 (NRG-hu Thy/HSC/HIV-1/cART/mIgG2a), *n* = 4 (NRG-hu Thy/HSC/HIV-1/cART/α-IFNAR1) mice. Each dot represents one individual mouse; bars indicate mean (*P < 0.05, **P < 0.01, and ***P < 0.001, by one-way analysis of variance and Bonferroni’s *post hoc* test).

We next determined whether IFNAR blockade can reverses aberrant immune activation and exhaustion phenotype of human T cells in both NRG-hu HSC and NRG-hu Thy/HSC mice. We found that in both HIV-1 infected NRG-hu HSC and NRG-hu Thy/HSC mice, cART alone significantly rescued the number of human CD4 and CD8 T cells (Figures 8A,B), however, it only slightly decreased the expression level of CD38/HLA-DR (Figures 8C,D) and PD-1 on CD8 T cells (Figures 8E,F). CD8 T cells from both cART-treated NRG-hu HSC and NRG-hu Thy/HSC mice still expressed significantly higher levels of activation marker (Figures 8C,D) and exhaustion marker PD-1 (Figures 8E,F) compared with uninfected hu-mice. Interestingly, IFNAR blockade significantly reversed aberrant CD8 T-cell activation and exhaustion in the presence of cART in both NRG-hu HSC and NRG-hu Thy/HSC mice (Figures 8C–F).

### IFNAR Blockade Reduce HIV-1 Reservoirs in Both HIV-1 Infected NRG-hu HSC and NRG-hu Thy/HSC Mice Under cART

**Figure 9:**
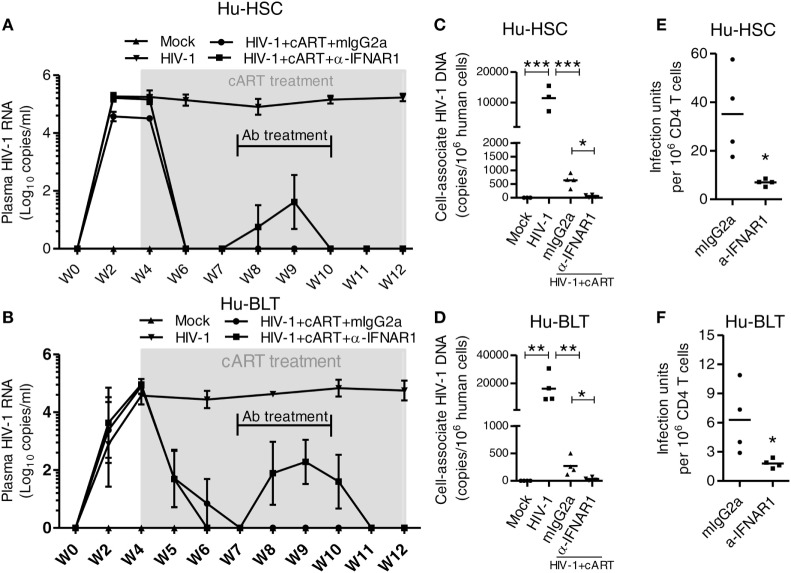
IFNAR blockade reduce HIV-1 reservoir in both HIV-1 infected NRG-hu HSC and NRG-hu Thy/HSC mice under combined antiretroviral therapy (cART). NRG-hu HSC and NRG-hu Thy/HSC mice infected with HIV-1 were treated with cART from 4 to 12 weeks postinfection (wpi). From 7 to 10 wpi, the cART-treated mice were injected with α-IFNAR1 antibody or isotype control mIgG2a antibody. **(A,B)** HIV-1 genomic RNA levels in the plasma of NRG-hu HSC **(A)** and NRG-hu Thy/HSC **(B)** mice. **(C,D)** Cell-associated HIV-1 DNA in human cells from spleen of NRG-hu HSC **(C)** and NRG-hu Thy/HSC **(D)** mice was quantified by PCR. **(E,F)** Replication-competent HIV-1 viruses from unfractionated human CD45^+^ cells from spleen of NRG-hu HSC **(E)** and NRG-hu Thy/HSC **(F)** mice were detected by the quantitative virus outgrowth assay. The frequency was determined by maximum likelihood statistics. The infectious units in CD4 T cells were calculated based on the percentage of CD4 T cells in total human CD45^+^ cells. Shown are representative data from *n* = 3 (NRG-hu HSC/Mock), *n* = 3 (NRG-hu HSC/HIV-1), *n* = 4 (NRG-hu HSC/HIV-1/cART/mIgG2a), *n* = 4 (NRG-hu HSC/HIV-1/cART/α-IFNAR1), *n* = 4 (NRG-hu Thy/HSC/Mock), *n* = 4 (NRG-hu Thy/HSC/HIV-1), *n* = 4 (NRG-hu Thy/HSC/HIV-1/cART/mIgG2a), *n* = 4 (NRG-hu Thy/HSC/HIV-1/cART/α-IFNAR1) mice. Each dot represents one individual mouse; bars indicate mean (*P < 0.05, **P < 0.01, and ***P < 0.001, by one-way analysis of variance and Bonferroni’s *post hoc* test).

Combined antiretroviral therapy is able to suppress HIV-1 replication but does not eradicate HIV reservoir, which cause virus rebound after cART interruption. We have reported before that during chronic phase of HIV-1 infection in humanized mice, blockade of IFN-I signaling using a mAb targeting to IFN-I receptor (IFNAR) reduces the level of T cell activation, reverses T cell exhaustion, and improves HIV-specific CD8^+^ T cells (29). Most strikingly, we found that IFNAR blockade during cART administration markedly reduced HIV-1 reservoirs (29). Here we compared the effect of IFNAR blockade in HIV-1 reservoir reduction in NRG-hu HSC and NRG-hu Thy/HSC mice. We treated HIV-1-infected NRG-hu HSC and NRG-hu Thy/HSC mice that were fully cART-suppressed with α-IFNAR1 mAb for 3 weeks during 7–10 wpi (Figures 9A,B). Interestingly, IFNAR blockade led to low blips of HIV-1 replication, which returned to undetectable levels after stopping α-IFNAR1 mAb treatment, in the presence of cART in both NRG-hu HSC and NRG-hu Thy/HSC mice (Figures 9A,B). We next analyzed the HIV-1 reservoir size in lymphoid organs 2 weeks after IFNAR blockade in both NRG-hu HSC and NRG-hu Thy/HSC mice. We measured cell-associated HIV-1 DNA by PCR, and replication-competent HIV-1 by the quantitative virus outgrowth assay. We found that IFNAR blockade reduced cell-associated HIV-1 DNA by 10.8-fold in the spleen of NRG-hu HSC mice (Figure 9C) and by 7.9-fold in NRG-hu Thy/HSC mice (Figure 9D). More importantly and consistently, IFNAR blockade significantly reduced the size of replication-competent HIV-1 reservoirs measured by quantitative virus outgrowth assay in both NRG-hu HSC and NRG-hu Thy/HSC mice (Figures 9E,F).

Taken together, we conclude that both NRG-hu HSC and NRG-hu Thy/HSC mouse models are valuable tools for the study of HIV-1 replication, pathogenesis and therapeutics.

## Discussion

Humanized mice with human immune cells are highly relevant and robust models for HIV-1 study (6–8). The models are generated *via* transplantation of CD34^+^ HSCs and/or implantation of human tissue into immunodeficient mice. There are different humanized mouse models available as well as different means to prepare them (6, 7, 9). The degree of human immune system reconstitution can vary between different models, and between different batches of HSCs and/or tissue donors, and non-standardized operating procedure between laboratories. Also, HIV-1 infection, replication and HIV-1 induced pathology can vary between different models and dependant on which HIV-1 virus strain is used. These factors make researchers, especially those who have limited experiences on humanized mouse models difficult to decide which model to choose for their studies. Here we performed a comprehensive parallel comparison of systemic immune reconstitution and HIV-1 replication, HIV-1 induced pathology and their response to cART and immunotherapy between two humanized mouse models, the NRG-hu HSC and NRG-hu Thy/HSC models. We used NRG-hu HSC and NRG-hu Thy/HSC mice transplanted with HSCs without or with thymus fragment from same donors into same background of immunodeficient mice in our experiment to minimize the variation factors. Our results indicate that both NRG-hu HSC and NRG-hu Thy/HSC mice support significant level of human immune reconstitution and comparable level of HIV-1 replication, immunopathology and responses to ART and immune therapy.

We and others have reported that all the major human lymphoid and myeloid lineage cells are developed in both NRG-hu HSC mice and hu-BLT mice (10–20). However, no study has performed to parallelly compare the human immune reconstitution in these two models which transplanted with HSCs ± thymus from same donor into the same background of immunodeficient mice. In the NRG-hu Thy/HSC model, we co-transplanted CD34^+^ HSCs by intravenous injection within 3 h after thymus fragment (without fetal liver fragment) transplantation. Human thymic organoid developed under the kidney capsule in our NRG-hu Thy/HSC as well as reported in hu-BLT mice which indicated that the fetal liver/thymus “sandwich” are not essential for thymic organoid development. Progenitor cells derived from CD34^+^ HSCs can serve as the source of thymocyte progenitors. It should be noted that the fetal liver fragments co-transplanted with thymus fragments in the BLT or SCID-hu Thy/Liv (48) mice also provide mostly human HSC/progenitors and no other liver-related functions. Our results indicate that similar level of total human CD45^+^ cells were developed in peripheral blood, spleen, mLNs, and BM of both NRG-hu HSC and NRG-hu Thy/HSC mice transplanted with HSCs ± thymus from same donor. We also found that all the major human lymphoid and myeloid lineage including T, B, NK cells, monocytes/macrophages, mDC, and pDC were developed in both NRG-hu HSC mice and NRG-hu Thy/HSC mice. The major difference between these two models is that human T cells are predominantly developed in NRG-hu Thy/HSC mice due to more efficient T cell development in human thymus tissue (or xeno-reactive T cells) in NRG-hu HSC-Thy mice. This may lead to preferential reconstitution of T cells and reduced NK/monocyte/pDC engraftment in NRG-hu HSC-Thy mice (Figures 1 and 3).

Our results indicated that majority of human T cells from both NRG-hu HSC and NRG-hu Thy/HSC mice were with naïve phenotype and they responded similarly to mitogen stimulation. However, it is important to point out that human T cells can develop in the presence of human thymic epithelium, resulting in human HLA class I and class II restriction in NRG-hu Thy/HSC mice (14, 15, 40). While in NRG-hu HSC mice, human T cells are produced in the mouse thymus and presumed to be educated in the context of mouse MHC (10–13). To study human HLA-restricted immune response in NRG-hu HSC mice, an immune-compromised non-obese diabetic/SCID/IL2rg^−/−^ strain (NSG) with homozygous expression of HLA class I heavy chain and light chain (NSG-HLA-A2/HHD) was generated (49). Human CTLs developing in the NSG-HLA-A2/HHD mice recognized EBV-derived peptides in an HLA-restricted manner and showed HLA-restricted cytotoxicity against EBV-infected human B cells (49). We also reported that HIV-1 infection can induce HIV-1 antigen-specific, HLA-A2-restricted CD8 T cell responds in humanized NSG-HLA-A2/HHD mice (31).

Both NRG-hu HSC and NRG-hu Thy/HSC mice support HIV-1 replication *in vivo*. Our results show that plasma HIV-1 viremia reached to peak levels at 2 wpi in NRG-hu HSC mice, while the peak viremia appeared at 4 wpi in NRG-hu Thy/HSC mice. The results suggest that anti-HIV-1 immunity at the early stage of HIV-1 infection is better in NRG-hu Thy/HSC mice. The better HLA-restricted anti-HIV-1 T cells response in NRG-hu Thy/HSC mice (23) may contribute to the delay of peak viremia. However, other unknown factors, such as the difference in immune subset reconstitution or donor genetics, may also lead to the reduced or delayed HIV-1 infection in NRG-hu Thy/HSC mice. After 4 weeks, HIV-1 replicated to similar levels in NRG-hu HSC and NRG-hu Thy/HSC mice. Furthermore, HIV-1 infection induced similar pathology including the depletion of human T cells and activation and exhaustion of T cells.

Combined antiretroviral therapy is able to suppress HIV-1 replication but does not eradicate HIV-1 reservoir, which cause virus rebound after cART interruption. This lack of *in vivo* models of HIV-1 infection has hindered progress in finding a cure for HIV-1/AIDS. The use of both NRG-hu HSC and NRG-hu Thy/HSC models of HIV-1 infection have made significant contribution to the field of HIV cure research (6, 7). We found here that both the HIV-1 infected NRG-hu HSC and NRG-hu Thy/HSC mice responded similarly to cART. Importantly, we found that type-I IFN signaling contributed to HIV-1 induced immune activation, dysfunction and fostered viral persistence in both NRG-hu HSC and NRG-hu Thy/HSC mice. Blockade of IFNAR reduced the level of T cell activation, reversed T cell exhaustion, and reduced HIV-1 reservoirs in both models. Multiple mechanisms may lead to the reduction of HIV-1 reservoir size after IFNAR blockade. The rescued human T cells could target the HIV-1 reservoirs with elevated gene expression and clear the reservoir cells as we have reported (29, 30). Other factors, including HIV-1 induced death of reservoir cells, reduced general T cell activation after IFNAR blockade, may also contribute to the reduction of HIV-1 reservoir size (29).

Taken together, we conclude that both NRG-hu HSC and NRG-hu Thy/HSC mouse models are relevant and robust for the study of HIV-1 replication, pathogenesis and therapeutics. Each model has its own advantage and disadvantages. Compared with the NRG-hu Thy/HSC or Hu-BLT models, the advantages of the NRG-hu HSC model are as follows: (1) the procedure to construct NRG-hu HSC mice is simple, which only involving pre-irradiating the neonate immunodeficient mice followed by injecting human CD34^+^ HSCs (10, 11, 13). To generate NRG-hu Thy/HSC or Hu-BLT mice, a time consuming and technically difficult surgery procedure is needed to implant the human thymus tissue under the kidney capsule of the mice (14, 15); (2) the source of HSCs to construct NRG-hu HSC mice is not restricted to fetal liver derived CD34^+^ cells. CD34^+^ HSCs from cord blood or human BM can also support the systemic development of human immune system (10, 14); (3) the graft-versus-host disease (GVHD) rarely happens in NRG-hu HSC mice, while the incident of GVHD is high in NRG-hu Thy/HSC or BLT mice (50) probably due to the mature human thymocytes in the transplanted thymic fragments; and (4) neonate immunodeficient mice are used to generate NRG-hu HSC mice, while 6- to 8-week-old mice are used for NRG-hu Thy/HSC or BLT mice construction. As the time needed for human immune reconstitution is 12–16 weeks in both models, researchers can start their experiments with younger NRG-hu HSC mice. The hu-Thy/HSC or BLT model also has its own advantages. It was reported that NOD-SCID-BLT (not NSG-BLT) mice supported better gut-associated lymphoid tissue development (GALT) (51). The other advantage of NRG-hu Thy/HSC or BLT model or hu-BLT model is that it supports the study of human HLA class I and class II restricted T cell response because human T cells develop in the presence of human thymic epithelium (14, 15, 40). However, as discussed earlier, human MHC-restricted T cell response and therapies can be studied in NRG-hu HSC mice that transgenically express human HLA-genes (49).

## Ethics Statement

The project was reviewed by the University’s Office of Human Research Ethics, which has determined that this submission does not constitute human subjects research as defined under federal regulations [45 CFR 46.102 (d or f) and 21 CFR 56.102(c)(e)(l)]. All animal studies were carried out in accordance with the recommendations of NIH guidelines for housing and care of laboratory animals. The protocol and was approved by the University of North Carolina Institutional Animal Care and Use Committee (IACUC ID: 14-100).

## Author Contributions

LC and LS conceived the study and designed the experiments. LC, JM, and GL performed the experiments. LC performed the analyses. LC and LS interpreted the data, wrote the manuscript, and supervised the study. All the authors approved the final version.

## Conflict of Interest Statement

The authors declare that the research was conducted in the absence of any commercial or financial relationships that could be construed as a potential conflict of interest.
