# Supplementary material for: Humanized Mice Engrafted With Human HSC Only or HSC and Thymus Support Comparable HIV-1 Replication, Immunopathology, and Responses to ART and Immune Therapy
Source: Front Immunol. 2018 Apr 19;9:817. doi: 10.3389/fimmu.2018.00817 (PMC5916969; doi:10.3389/fimmu.2018.00817)
Supplement: Supplementary file 1 [file Presentation_1.PPTX]

## Slide 1
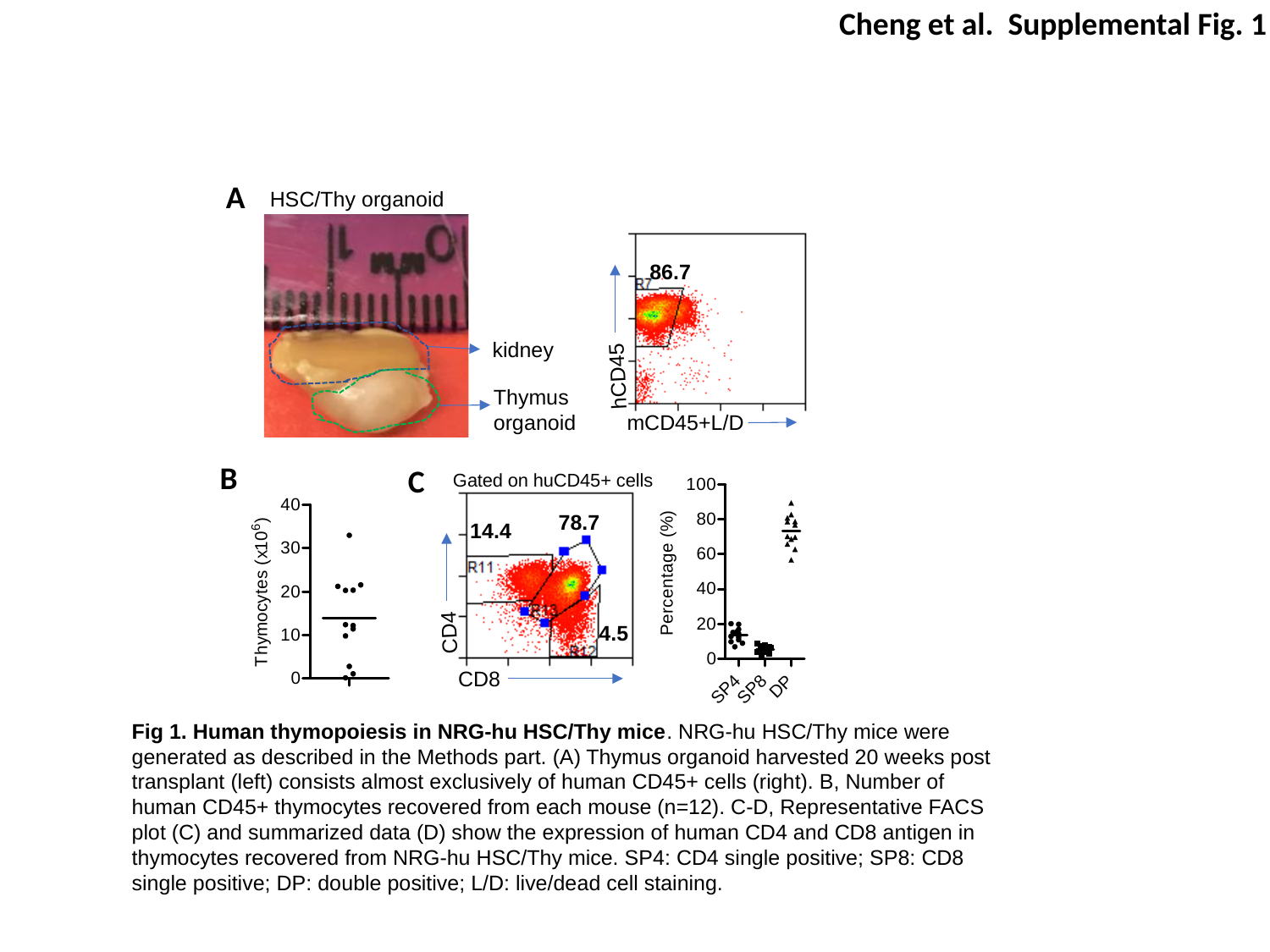

Cheng et al. Supplemental Fig. 1
A
HSC/Thy organoid
86.7
hCD45
mCD45+L/D
kidney
Thymus
organoid
C
B
Gated on huCD45+ cells
78.7
14.4
CD4
4.5
CD8
Fig 1. Human thymopoiesis in NRG-hu HSC/Thy mice. NRG-hu HSC/Thy mice were generated as described in the Methods part. (A) Thymus organoid harvested 20 weeks post transplant (left) consists almost exclusively of human CD45+ cells (right). B, Number of human CD45+ thymocytes recovered from each mouse (n=12). C-D, Representative FACS plot (C) and summarized data (D) show the expression of human CD4 and CD8 antigen in thymocytes recovered from NRG-hu HSC/Thy mice. SP4: CD4 single positive; SP8: CD8 single positive; DP: double positive; L/D: live/dead cell staining.

## Slide 2
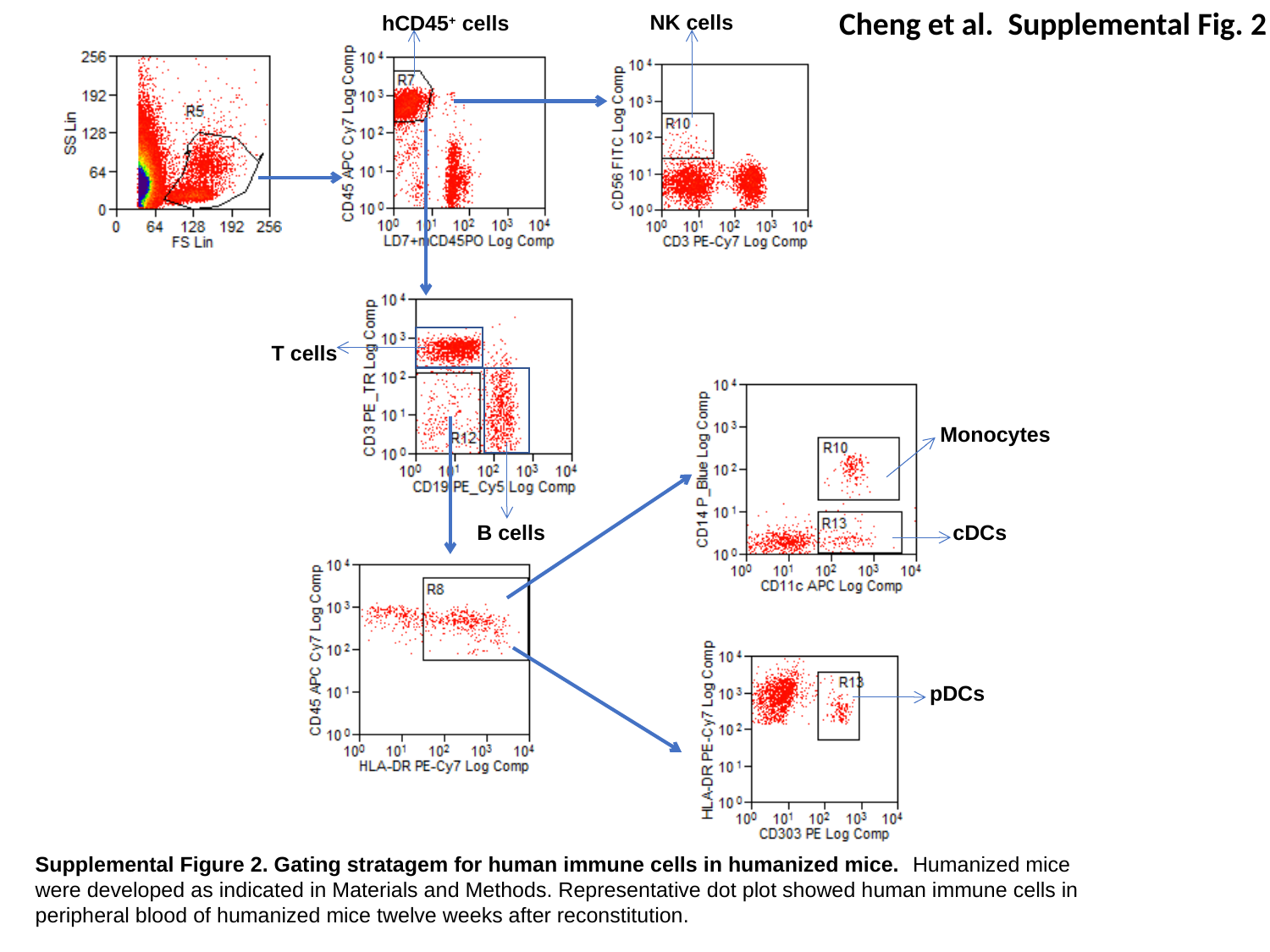

Cheng et al. Supplemental Fig. 2
NK cells
hCD45+ cells
T cells
Monocytes
cDCs
B cells
pDCs
Supplemental Figure 2. Gating stratagem for human immune cells in humanized mice. Humanized mice were developed as indicated in Materials and Methods. Representative dot plot showed human immune cells in peripheral blood of humanized mice twelve weeks after reconstitution.

## Slide 3
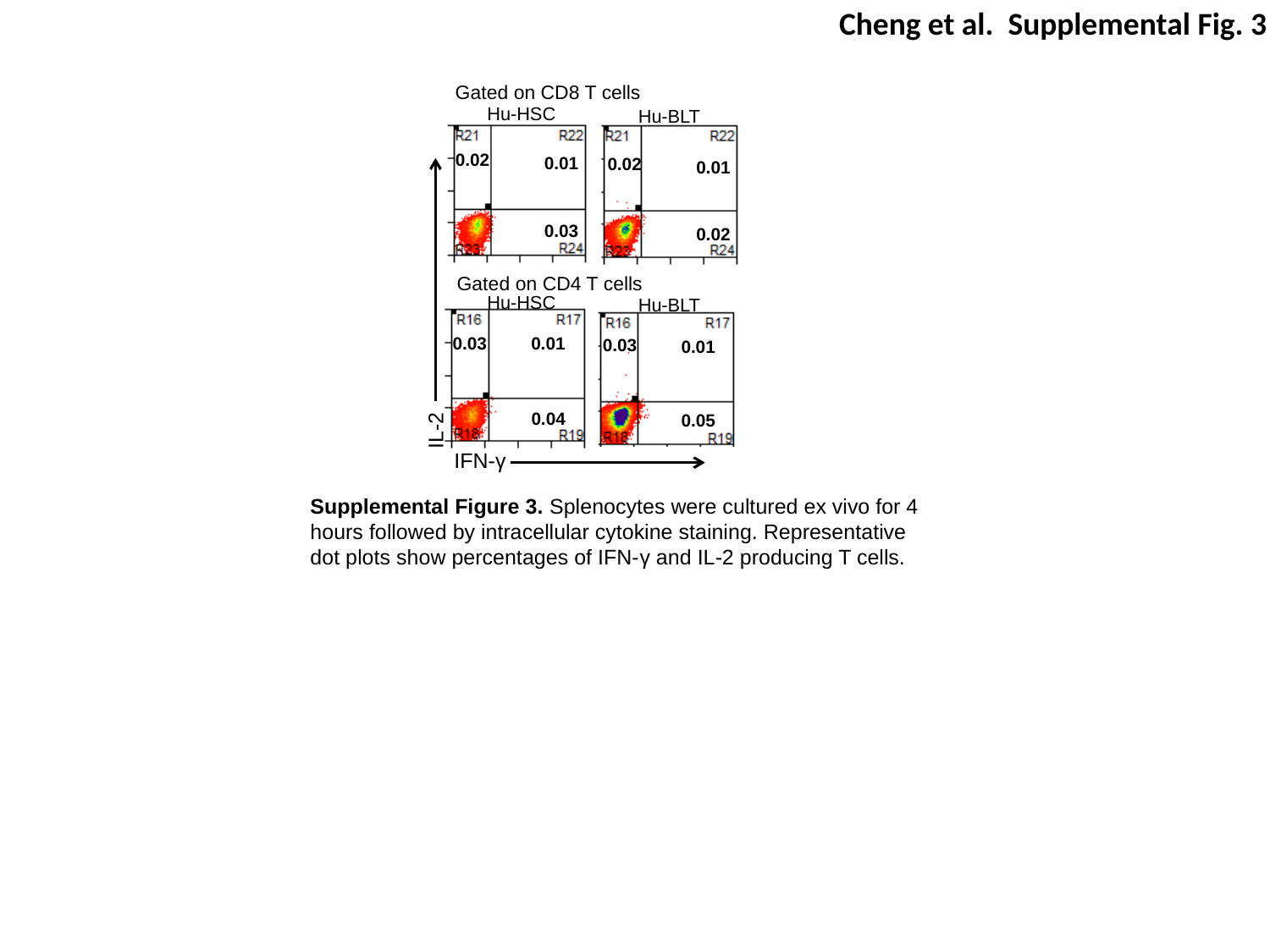

Cheng et al. Supplemental Fig. 3
Gated on CD8 T cells
Hu-HSC
Hu-BLT
0.02
0.01
0.02
0.01
0.03
0.02
Gated on CD4 T cells
Hu-HSC
Hu-BLT
0.01
0.03
0.03
0.01
0.04
0.05
IL-2
IFN-γ
Supplemental Figure 3. Splenocytes were cultured ex vivo for 4 hours followed by intracellular cytokine staining. Representative dot plots show percentages of IFN-γ and IL-2 producing T cells.
